# Evaluation of the extent of neoplastic infiltration in small intestinal tumours in dogs

**DOI:** 10.1002/vms3.147

**Published:** 2019-02-19

**Authors:** Michael Morrice, Gerry Polton, Sam Beck

**Affiliations:** ^1^ Maven Veterinary Care London UK; ^2^ North Downs Specialist Referrals Bletchingly UK; ^3^ Bridge Pathology Ltd. Bristol UK

**Keywords:** canine, intestine, margin, surgery, tumour

## Abstract

There is currently a lack of evidence‐based guidance when determining surgical margins for small intestinal tumours in dogs. The purpose of this study is to help the surgeon make informed clinical decisions about margins when confronted with a small intestinal mass. Twenty‐seven canine small intestinal tumours were histologically diagnosed and then had further histological assessment at every centimeter from the edge of the palpable tumour to the surgical margin in oral, aboral and mesenteric directions. In all 10 carcinomas a 3 cm tissue margin in oral, aboral and mesenteric directions would have resulted in complete tumour resection. In all 11 sarcomas a 2 cm tissue margin in oral, aboral and mesenteric directions would have resulted in complete tumour resection. Five of the six intestinal lymphomas would have required tissue margins of 4 cm or more for complete resection. Of the 21 non‐lymphoma tumours assessed in this study, complete resection was achieved in all 21 (100%) with tissue margins at 3 cm from the palpable edge of the mass, 20 (95%) with tissue margins at 2 cm from the palpable edge of the mass, and 16 (76%) with tissue margins at 1 cm from the palpable edge of the mass in oral and aboral directions. All non‐lymphoma canine small intestinal masses will be completely resected when tissue margins are 3 cm from the palpable edge of the mass in oral and aboral directions after fixation in formalin.

## Introduction

Alimentary tumours are uncommon in dogs and make up approximately 8% of tumours in this species (Crawshaw *et al*. 1998; Dobson *et al*. 2002). There are four general tumour categories that occur within the canine intestine. These include epithelial, mesenchymal, neuroendocrine and round cell neoplasms (Selting 2013) with half of all gastrointestinal tumours in the dog being adenocarcinomas (White 2003).

Surgery is the recommended treatment option for intestinal masses with the current exception of lymphoma (Shales 2015). Lymphoma is usually treated with chemotherapy unless there is perforation, the need for a biopsy or intestinal obstruction (Culp *et al*. 2012; Selting 2013). There is, however, growing evidence in both veterinary and human literature supporting the combination of surgery with chemotherapy to treat discrete intestinal lymphoma (Kim *et al*. 2011; Gou *et al*. 2012; Gouldin *et al*. 2017; Hong *et al*. 2017).

When considering surgery for solitary discrete intestinal neoplasia there are currently a wide range of recommendations for surgical margins in both the small and large intestine (Table 1). These recommendations are mostly based on expert opinion rather than data. Importantly, in both the veterinary and human literature, survival time has been shown to be strongly influenced by the presence or absence of complete or incomplete surgical margins (Slawienski *et al*. 1997; Bakaeen *et al*. 2000; Zhang *et al*. 2011).

**Table 1:** This table summarises the current available recommendations for surgical margins when treating solitary intestinal neoplasia in dogs and cats

Source	Intestinal margin recommendation
Crawshaw *et al*. 1998;	5 cm of bowel on either side of the tumour and wide mesenteric resection.
Tumours of the gastrointestinal tract and associated structures. In Small Animal Oncology: An Introduction (North & Banks 2009)	4–8 cm
Marks 1996	At least 4 cm
Tumours of the colon and rectum. In BSAVA Manual of Canine and Feline Oncology Third Edition (Bray^2011^)	2–8 cm for colorectal neoplasia
Alimentary Tract. In Veterinary Surgical Oncology (Culp *et al*. 2012)	5 cm
BSAVA Manual of Canine and Feline Oncology Second Edition (White 2003)	Wide local resection with margins extending 4–8 cm.
Morello *et al*. 2008	5 cm for colorectal tumours

The aim of this study was to evaluate the extent of tumour infiltration in the small intestine of dogs. By assessing the intestine and mesentery adjacent to the grossly appreciable neoplasm in a similar manner to a previous veterinary study on canine cutaneous mast cell tumours (Simpson *et al*. 2004) it was hoped that data could be generated that could contribute to the future development of surgical guidelines for these tumours.

## Methods and materials

This study was designed as a prospective study. Single discrete small intestinal tumours removed at veterinary centres across the United Kingdom and Ireland from March 2017 to March 2018 that were sent to Bridge Pathology Limited (www.bridgepathology.com) for histopathological assessment were collected post diagnosis for further investigation by the author having been fixed in formalin. All intestinal tumours assessed by Bridge Pathology Limited in this period were included in the study if there was sufficient margin left for assessment after fixing, processing and diagnostic sampling.

**Figure 1:**
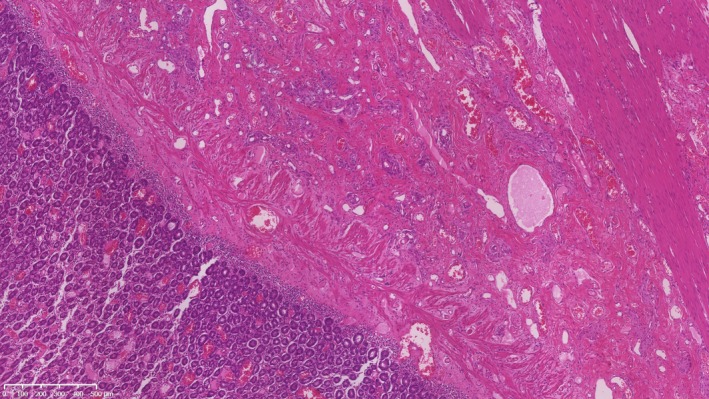
A canine intestinal carcinoma.

Twenty‐seven masses were tested further to determine how much tissue would need to be taken in the oral, aboral and mesenteric directions to achieve complete tumour resection in each case. When assessing the intestine, a transverse sample was taken at the palpable tumour edge and then every 1 cm from this edge to the closest 1 cm increment to the surgical margin. The surgical margin for each mass was therefore always within 1 cm of the most distal centimetre in this study. The amount of tissue available for assessment varied from case to case. Because the aboral and oral directions were not known by the author, one direction was termed the left side, the other the right side. Where available, the mesentery was assessed as per the intestine starting at the tumour's palpable border and then at every 1 cm from the tumour in a direction perpendicular to the intestine. The area selected along the length of the tumour (parallel to the intestine) for mesenteric sampling was at the tumour's oral or aboral limits rather than centrally due to initial diagnostic sections having already been taken from this site. Sampled tissue was then embedded in paraffin wax and cut at 4–5 microns before floating onto glass slides as previously described (Beck *et al*. 2011). These sections were routinely deparaffinised, rehydrated and stained with haematoxylin and eosin (HE) using a Gemini AS automated slide stainer (ThermoFisher Scientific, Cheshire, UK). The individual sections were examined by a single board certified pathologist (Sam Beck BSc BVSc MVetMed FRC‐Path MRCVS Dip.ACVP) to determine the presence or absence of neoplasia (Fig. 1).

Immunohistochemistry was performed in a low number of cases (see Data S1) following examination of the HE stained tissue. Depending on the differential diagnoses the following primary antibodies were used: vimentin (Dako, 1:2000), cytokeratin AE1/AE3 (Dako, 1:400), CD18 (Leukocyte Antigen Lab, 1:20), CD3 (Dako, 1:400), CD20 (Dako 1:400), Pax‐5 (Dako, 1:160), MUM‐1 (Dako, 1:50), CD117 (Dako, 1:400), S100 (Dako, 1:1000), desmin (1:200) and smooth muscle actin (Dako 1:500). Briefly, 5‐micron sections of each neoplasm were cut onto positively charged glass slides and loaded into an automated staining system (Dako Autostainer Link 48). Antigen retrieval was performed at pH 9 and samples were incubated with the primary antibody. Labelling of primary antibody binding was performed via Envision secondary labelling and chromogen DAB reaction (Dako Envision Flex Link). Positive control blocks were used for each sample. Endogenous peroxidase was blocked via Envision peroxidase solution (Dako). Slides were counterstained with haematoxylin.

Leiomyosarcomas, fibrosarcomas and gastrointestinal stromal tumours have been grouped together under the heading of sarcomas.

All six small intestinal lymphoma tumours were initially diagnosed by routine histology; immunohistochemistry testing was performed in only one lymphoma case. Each centimeter segment in the study was assessed by routine histology only.

For each tumour a left‐sided, right‐sided and mesenteric measurement was determined that would have resulted in complete resection of that individual tumour. Tumours were then grouped into carcinomas, sarcomas and lymphomas. Based on the collective surgical margins of each group, recommendations were then determined for surgical margins for canine intestinal tumours of the individual tumour types above and for intestinal tumours collectively.

## Results

Of the 27 small intestinal tumours examined in this study, 10 were identified as carcinomas, 11 were sarcomas and six were lymphomas. All tumours were located within the small intestine. The location could be defined more accurately in 21 cases to the duodenum (eight), the jejunum (11) or the ileum (one).

For each neoplasm the left‐sided, right‐sided and mesenteric surgical margins that would have resulted in complete tumour excision, the dog's signalment, the intestinal tumour type, the mitotic index, evidence for spread to local lymph nodes (where available) and other evidence for metastatic spread (where available) are recorded in Table 2. The initial diagnostic pathology reports for all cases are also included in Data S1.

**Table 2:** This table contains the results of this study's research. Within the table is information about each of the 27 small intestinal tumours including the resection margins required for complete removal of each of these masses

Case No	Breed	Age	Signalment	Tumour Location	Histological diagnosis	Mitotic index	Lymph node metastasis	Other metastatic spread	Intestinal margin required for complete removal left hand side	Intestinal margin required for complete removal right hand side	Mesenteric margin required for complete removal
3	Boxer	10 years, 6 months	M	Jejunum	Carcinoma	35 per 10xHPF	Unknown	Unknown	1 cm	1 cm	0 cm
20	Samoyed	12 years old	FN	Duodenum	Carcinoma	4 per 1xHPF	Unknown	Unknown	2 cm	1 cm	0 cm
21	Staffordshire Terrier	14 years old	FN	Small Intestine	Carcinoma	21 per 10xHPF	Unknown	Unknown	0 cm	1 cm	0 cm
22	Labradoodle	11 years old	FN	Duodenum	Adenocarcinoma	5 per 1xHPF	Yes	Unknown	0 cm	0 cm	0 cm
15	Cocker Spaniel	7 years, 9 months	M	Duodenum	Carcinoma	12 per 10xHPF	Yes	Unknown	0 cm	1 cm	1 cm
12	Corgi	5 years old	F	Ileum	Carcinoma	5 per 1xHPF	Unknown	Unknown	1 cm	2 cm	1 cm
13	Bulldog	11 years, 7 months	MN	Jejunum	Adenocarcinoma	2 per 10xHPF	Unknown	Unknown	0 cm	>2 cm	1 cm
6	Staffordshire Terrier	12 years old	FN	Jejunum	Adenocarcinoma	41 per 10xHPF	No	Unknown	1 cm	1 cm	0 cm
1	Border Terrier	13 years old	M	Jejunum	Carcinoma	6 per 10xHPF	Unknown	Unknown	2 cm	0 cm	2 cm
24	Jack Russell	8 years, 2 months	MN	Duodenum	Adenocarcinoma	37 per 10xHPF	No	Unknown	1 cm	0 cm	0 cm
10	Labrador	11 years, 9 months	MN	Small Intestine	Leiomyosarcoma	6 per 10xHPF	Unknown	Unknown	1 cm	0 cm	0 cm
11	Eurasian	10 years, 11 months	MN	Small Intestine	Fibrosarcoma	18 per 10xHPF	Unknown	Unknown	0 cm	0 cm	n/a
14	Labrador	10 years, 0 months	FN	Duodenum	Gastrointestinal stromal tumour	0 per 10xHPF	Unknown	Unknown	0 cm	0 cm	1 cm
16	Border Terrier	7 years old	M	Jejunum	Leiomyosarcoma	4 per 10xHPF	Unknown	No	0 cm	1 cm	0 cm
17	Lurcher	9 years, 7 months	MN	Jejunum	Sarcoma	2 per 10xHPF	Unknown	No	0 cm	2 cm	0 cm
4	American Staffordshire Terrier	10 years, 0 months	MN	Duodenum	Leiomyosarcoma	58 per 10xHPF	Unknown	Unknown	0 cm	0 cm	0 cm
5	Cross breed	6 years, 9 months	FN	Duodenum	Gastrointestinal stromal tumour	5 per 10xHPF	Unknown	Unknown	0 cm	0 cm	>0 cm
7	Greyhound	9 years, 10 months	MN	Small Intestine	Leiomyosarcoma	7 per 10xHPF	Unknown	Unknown	0 cm	0 cm	n/a
8	Cross breed	6 years, 8 months	FN	Small Intestine	Sarcoma	13 per 10xHPF	Unknown	Unknown	1 cm	0 cm	n/a
27	Springer Spaniel	9 years, 5 months	M	Jejunum	Leiomyosarcoma	3 per 10xHPF	No	Unknown	0 cm	0 cm	0 cm
23	Yorkshire Terrier	10 years, 0 months	MN	Jejunum	Sarcoma	2 per 10XHPF	Unknown	No	0 cm	1 cm	0 cm
2	Yorkshire Terrier	7 years old	MN	Small Intestine	Lymphoma	16 per 10xHPF	Unknown	Unknown	8 cm	2 cm	0 cm
9	Cross breed	10 years, 9 months	FN	Jejunum	Lymphoma	11 per 1xHPF	Yes	No	>4 cm	>2 cm	0 cm
18	Greyhound	10 years, 5 months	FN	Jejunum	Lymphoma	6 per 1xHPF	Yes	Unknown	>6 cm	3 cm	0 cm
19	Lhapso Apso	7 years, 0 months	M	Small Intestine	Lymphoma	24 per 10xHPF	No	Unknown	4 cm	Only one margin	0 cm
25	Cross breed	4 years, 0 months	FN	Duodenum	Lymphoma	11 per 1xHPF	Unknown	Unknown	0 cm	1 cm	0 cm
26	Shih Tzu	9 years, 3 months	MN	Jejunum	Lymphoma	6 per 1xHPF	Unknown	Unknown	6 cm	0 cm	0 cm

There were a variety of breeds noted over the 27 cases with no clear breed predilection. Both males and females were similarly represented. The median age of all dogs was 10 years of age. The median age of dogs with carcinomas was 11 years, sarcomas was 10 years, and lymphomas was 8 years.

Of the 10 small intestinal carcinomas an expansile lesion was noted in two cases. These were 30 × 40 mm and 40 × 80mm in size. The remaining carcinomas were annular in type. The mitotic index ranged from 2 per 10 high‐powered fields to 5 per single high‐powered field over the 10 cases. The two expansile carcinomas (cases 15 and 20) had a mitotic index of 12 per ten high‐powered fields and 4 per single high‐powered field, respectively. The carcinoma (case 13) with the lowest mitotic index required the widest margins for complete resection. When assessing the carcinomas, nine of 10 would have been completely resected with 2 cm margins in oral, aboral and mesenteric directions. One carcinoma (case 13, Table 2) would have required a 3 cm margin for complete resection because the surgical margin on the right hand side of this tumour was noted to be free of neoplastic cells in the diagnostic histopathology report despite there being neoplastic cells at the 2 cm section in this study.

Of the 11 sarcomas, four were leiomyosarcomas, two were gastrointestinal stromal tumours and one was a fibrosarcoma. Four sarcomas did not have immunohistochemistry performed. There was an expansile mass noted grossly in seven on the 11 cases with tumour size ranging from 15 by 15 mm to 40 mm by 40 mm. Over the 11 cases the mitotic index ranged from 0 to 58 mitosis per 10 high‐powered fields. There was no correlation noted between mitotic index and required surgical margin for complete resection in this group. For example, the three cases with the highest mitotic index (58, 18 and 13 per 10 high‐powered fields) required surgical margins of 1 cm or less in all directions for complete tumour resection. All of the 11 sarcomas would have been completely resected in oral, aboral and mesenteric directions with margins of 2 cm. Ten of 11 sarcomas would have been completely resected with 1 cm margins. The only possible exception is noted in case number five where neoplasia was noted at the 0 cm mesenteric margin and the 1 cm margin was not available for assessment.

If the carcinomas and sarcomas are grouped together and margins in oral, aboral and mesenteric directions are assessed: all 21 (100%) tumours would have been completely removed with 3 cm margins; 20 of the 21 (95%) would have been completely removed with margins of 2 cm; and 16 of the 21 (76%) tumours would have been completely removed with margins of 1 cm.

Of the six small intestinal lymphoma cases reported two were high grade, two were intermediate grade and two were low grade (Valli *et al*. 2011). An expansile mass was noted grossly in five of six cases with tumour size ranging from 25 × 35 mm to 70 × 90 mm. Over the six cases the mitotic index ranged from 16 mitosis per 10 high‐powered fields to two cases with 11 mitosis per single high‐powered field. Of the cases examined, one of the two lymphomas with the highest mitotic index would have been completely excised with margins of 1 cm, while the other would have needed margins in excess of 4 cm in oral, aboral and mesenteric directions. The lymphoma with the lowest mitotic index required a surgical margin 8 cm for complete tumour resection. There was comparative variation when assessing the margins of the six intestinal lymphomas. In one of these cases a 1 cm margin in oral, aboral and mesenteric directions would have resulted in complete tumour resection. There were three intestinal lymphomas with neoplastic cells present 6 cm from the palpable edge of the intestinal mass. In cases 2 and 18 there was segmental infiltration of the intestine away from the primary mass.

Of the 27 canine small intestinal tumours assessed, the mesenteric margin was available for surgical margin determination in 24 cases. Of these, all mesenteric margins would have been tumour free at 2 cm from the palpable tumour edge, 23 would have been tumour free 1 cm from the palpable tumour edge and in 18 of the 24 tumours there was no evidence for any mesenteric invasion. In cases 6, 22 and 24 the initial diagnostic histopathology report describes invasion of neoplastic cells into the mesentery. This was not found in this study's examined sections.

Biopsies from regional lymph nodes were available in eight of the 27 tumours. There was confirmed metastatic spread to these nodes in two carcinomas and two lymphoma cases.

## Discussion

The results of this study suggest that for non‐lymphoma, formalin fixed, small intestinal tumours a 3 cm margin in oral and aboral directions would result in complete resection of the tumour.

Complete resection of intestinal tumours is important. In one human study of duodenal neoplasia, lymph node metastasis as well as positive resection margins had a significantly negative impact on survival times in patients undergoing potentially curative surgery (Zhang *et al*. 2011). In another human study of duodenal adenocarcinomas, the 3‐ and 5‐year actuarial survival rates were 64% and 58% respectively for patients with clear surgical margins and 38% and 25% for those with positive margins. This study also showed that as long as a clean surgical margin can be secured there is no difference in survival times between those patients undergoing radical resection to those undergoing limited resection (Bakaeen *et al*. 2000). In the veterinary literature there is a wide variety of recommendations when considering surgical margins for intestinal tumour removal in the dog. To date, these recommendations are based on expert opinion rather than data (Shales 2015).

There are few recommendations in the literature for mesenteric margin resection for intestinal tumours. One expert opinion is for wide mesenteric margins (Crawshaw *et al*. 1998). Of the 24 available mesenteric margins assessed in this study, complete tumour resection would have been achieved in all with margins at 2 cm. Importantly there are three cases in this study where no neoplastic mesenteric invasion was noted either grossly by the author or in the examined mesenteric sections in this study despite a histological description to the contrary in the diagnostic histopathology reports. This is because the samples harvested for examination in the study were obtained from the palpable oral and aboral limits of the tumour (and then at every 1 cm perpendicular to the tumour) rather than centrally within the tumour, as was the case for the diagnostic samples. This difference due to the variability in the area of the mesentery sampled should serve as a warning to the reader that the results of this study may require validation in a larger study before being adopted.

Clarke *et al*. (2014) reported that the canine small intestine length will contract by 28.3% immediately after excision and by 26.3% after 24 h in formalin. In a human study, it was shown that the small intestine length would contract by 21.8% and the large intestine by 36.4% after 12 h in formalin but would not contract further beyond the 12‐h mark (Wang *et al*. 2004). Wang *et al*. (2004) also showed that under conditions where the surgeon put between 500 and 1000 g of pull force on the intestine during surgery, the small intestine stretched by 66.4% and 120%, respectively and the large intestine by 36% and 56%. Another human article discusses that the constitution and type of tissue may influence the degree of tissue shrinkage after formalin fixation. It also found that the average shrinkage of head and neck tumours after fixation was only 4.4% (Chen *et al*. 2012). As a result, when considering the margins recommended by this study, adjustment is needed to accommodate for this tissue shrinkage artifact. The degree of shrinkage in this study's samples may also be effected by the presence of neoplasia. When measuring margins for intestinal tumours *in vivo*, the surgeon should not put tension on the intestinal tissue so as to not inadvertently alter the planned tissue margins.

The margins required for complete tumour resection in the sarcoma group were similar regardless of the subtype of sarcoma. In one study that looked to re‐classify previously diagnosed leiomyosarcomas as gastrointestinal stromal tumours no significant difference was found in median survival times between dogs with leiomyosarcomas and those with gastrointestinal stromal tumours unless dogs dying in the immediate postoperative period were removed from the study (Russell *et al*. 2007). Another study concluded that the prognosis for intestinal tumours that could be classified as either leiomyomas, leiomyosarcomas, gastrointestinal stromal tumours or gastrointestinal stromal tumour like tumours is good after complete resection and not related to tumour type or location (Maas *et al*. 2007). Therefore it is likely that differentiation of these tumours beyond sarcomas would not alter the recommendations or results of this study.

In this study, five of the six intestinal lymphomas had segmental and widespread neoplastic involvement throughout the sectioned intestine. In the majority of cases, neoplastic cells extended well beyond the palpable mass. These results show that canine discrete small intestinal lymphoma is unlikely to be resolved with surgery alone and supports the notion that lymphoma is typically treated medically (Giuffrida & Brown 2018). A recent veterinary publication supports the combination of surgery and chemotherapy to treat feline intestinal lymphoma (Gouldin *et al*. 2017). This treatment combination has also been shown to improve overall survival of people treated for intestinal lymphoma (Kim *et al*. 2011; Gou *et al*. 2012). Surgical treatments are performed as the initial treatment followed by chemotherapy or radiation therapy if necessary. In people, gross resection of the main lesion should be prioritised over achieving margin‐free status (Hong *et al*. 2017).

Only eight of the 27 intestinal tumours in this study had mesenteric lymph node biopsy at the time of surgery despite this being recommended in the literature (Crawshaw *et al*. 1998). Of the lymph nodes biopsied, four were in intestinal carcinoma cases, one was in a sarcoma case, and three were in lymphoma cases. Of the sarcoma and carcinoma cases with lymph node biopsy, there was lymph node metastasis in two cases. The tumours in these cases could have been removed in their entirety with 1 cm margins in oral, aboral and mesenteric directions. Therefore, there appeared to be no correlation between requiring a larger intestinal surgical margin and lymph node metastasis in these two cases.

The main limitation of this study is the small number of cases featured. This is especially so in the sarcoma group where sub‐categorisation can differentiate these tumours further. There is also no follow‐up reported in any of the cases. Additional prospective studies are warranted to confirm the accuracy of the results reported here.

Despite the small number of cases, there is consistency in evidence of infiltration in the non‐lymphoma intestinal tumours. After fixation in formalin, all 21 (100%) assessed in this study would have been completely resected with margins at 3 cm from the palpable edge of the mass, 20 (95%) would have been completely resected with margins at 2 cm from the palpable edge of the mass, and sixteen (76%) would have been completely resected with surgical margins at 1 cm from the palpable edge of the mass. Preservation of the distal rectum, the biliary and pancreatic ducts and where possible, the ileo‐caecal junction, are important surgical considerations for resection of intestinal tumours (White 2003; Gorman *et al*. 2006; Morello *et al*. 2008). If anatomy limits intestinal tumour resection margins then the above observations may assist surgeons’ decision‐making.

## Source of funding

None declared.

## Conflicts of interest

None declared.

## Contributions

DC and the staff at Bridge Pathology Ltd provided help in the collecting and processing of the tissue samples in this study.

## Ethics statement

The authors confirm that the ethical policies of the journal, as noted on the journal's author guidelines page, have been adhered to. No ethical approval was required.

## Supporting information


**Data S1.** This appendix contains the Bridge Pathology initial diagnostic reports for all twenty‐seven small intestinal tumours in this study.
