# Supplementary material for: Evaluation of the extent of neoplastic infiltration in small intestinal tumours in dogs
Source: Vet Med Sci. 2019 Feb 19;5(2):189–98. doi: 10.1002/vms3.147 (PMC6498519; doi:10.1002/vms3.147)
Supplement: Supplementary file 1 — Data S1. This appendix contains the Bridge Pathology initial diagnostic reports for all twenty‐seven small intestinal tumours in this study. [file VMS3-5-189-s001.docx]

**Appendix 1**

**Histopathology Reports 1-27**

Case 1

**Clinical History:**

Intestinal mass (jejunal).

**Gross description:**

Jejunal mass.

A beige-brown section of tubular tissue measuring 150x35x35mm. Representative sections taken.

**Histological description:**

Jejunum (7 sections). At the site of the mass identified grossly, is a poorly demarcated, highly infiltrative, variably densely cellular, non-encapsulated neoplastic mass. Neoplastic cells are not apparent at the proximal distal surgeon-cut edges of the intestinal segment. Neoplastic cells extend from the ulcerated mucosal surface, throughout the muscularis layers and widely into the

mesentery. Neoplastic cells are arranged in dense nests, clusters and occasionally in acinar structures, supported by moderate amounts of fibrous connective tissue stroma. In many areas neoplastic cells are surrounded by abundant mucin. Individual neoplastic cells are polygonal, have variably distinct cell borders and contain moderate to large amounts of eosinophilic cytoplasm. Nuclei are oval to irregular oval and contain clumped to stippled chromatin with 1-2 moderately prominent nucleoli. Mitoses are 6 per 10 high power fields. There is a marked degree of anisokaryosis and anisocytosis throughout the neoplastic cell population. Scattered clusters of small lymphocytes are infiltrating amongst the neoplastic cells.

**Histological diagnosis:**

Carcinoma, poorly differentiated, small intestine.

**Comment:**

This mass represents a poorly differentiated carcinoma which is infiltrating widely throughout the muscularis layers, the submucosa and extensively within the adjacent mesentery. Given these findings, and although the mass does appear to have been fully excised with no neoplastic cells at the proximal or distal surgeon-cut ends of the intestinal segment, given this highly infiltrative growth pattern, there would be significant potential for metastasis from this site.

Case 2

**Clinical History:**

6 week history of mild intermittent ?? Suspect tumour in abdomen.

**Gross description:**

A beige-brown section of tubular tissue measuring 300x70x45mm with a mass measuring 95x70mm. Representative sections taken. Tissue fixed further prior to processing.

**Histological description:**

Small intestine (4 sections). Diffusely infiltrating and largely effacing the mucosa and extending into the submucosa and muscular wall is a poorly demarcated, unencapsulated, densely cellular neoplastic mass. Neoplastic cells are arranged in dense sheets infiltrating the pre-formed stroma. Individual neoplastic cells are round to oval with indistinct cell borders and moderate amounts of eosinophilic cytoplasm. Cells are often surrounded by a clear Harlow. Nuclei are

round to oval with stippled chromatin and predominantly indistinct nucleoli. Mitoses are 16 in 10 high power fields. Nuclei are 2-3x the size of adjacent erythrocytes. Admixed with the neoplastic cells are scattered low numbers of eosinophils. Variably, plasma cells, neutrophils and lymphocytes are present. In sections from the surgical margins, no neoplastic cells are evident.

**Histological diagnosis:**

Round cell tumour, small intestine.

**Comment:**

The mass present in the submitted sample of small intestine represents a round cell tumour with the main differential diagnoses of a lymphoma and a mast cell tumour. A plasmacytoma is considered unlikely. Immunohistochemistry could be performed to further characterise the neoplastic cells upon your request (6 antibodies). In sections from the proximal and distal surgeon-cut edge, no neoplastic tissue is evident, suggesting that complete local excision has

been achieved.

Case 3

**Clinical History:**

History of microcytic hyperchromatic anaemia, intermittent melena and haematochezia. Jejunal mass, enterectomy.

**Gross description:**

A beige section of tubular tissue measuring 90x45x22mm. Representative sections taken.

**Histological description:**

Jejunum (3 sections). The intestinal epithelium is multifocally extensively ulcerated. The underlying lamina propria and submucosa are multifocally infiltrated by a non-encapsulated neoplasm. Neoplastic cells are arranged in sheets and occasional nests supported by abundant fibrovascular stroma. Neoplastic cells are polygonal to spindloid with a moderate amount of

eosinophilic cytoplasm and indistinct cell borders. Nuclei are oval with finely stippled chromatin and 1-2 nucleoli. There is moderate anisocytosis and anisokaryosis. 35 mitoses are observed in 10 high power fields. Within the underlying tunica muscularis there are multifocal large aggregates of basophilic mucinous material. These are surrounded by a layer of attenuated spindloid cells. Within the stroma there are large numbers of lymphocytes, plasma cells and neutrophils. Within sections through the surgeon-cut tissue borders of the submitted sample, the mucosal architecture is preserved. There is multifocal mild oedema within the mucosa. No obvious neoplastic tissue is observed.

**Histological diagnosis:**

Poorly differentiated neoplasm, jejunum.

**Comment:**

The jejunal mass is a poorly differentiated neoplasm. In some areas the neoplastic cells form small nests and clusters and this may represent a poorly differentiated carcinoma. There are numerous spindle cells which may represent a reactive fibrous stroma of a carcinoma. However there is mitotic activity within the spindle cell population and this tumour could also represent a

poorly differentiated spindle cell neoplasm, such as a leiomyosarcoma or gastrointestinal stromal tumour. Immunohistochemistry can be performed on this sample on request for further characterisation of the neoplastic population (4 antibodies). There is no obvious neoplastic tissue within the surgeon-cut tissue borders of the submitted jejunal segment. Therefore based on

these sections complete excision appears to have been achieved. Nevertheless this tumour is expected to have potential for metastasis.

Case 4

**Clinical History:**
Duodenal mass 4-5 cm.  
Macroscopic Description:
A beige-brown section of tubular tissue measuring 155x40x30mm, with a mass measuring 40x40mm. Margins are inked on receipt of tissue. Representative sections taken.
**Microscopic Description:**
Duodenal mass (4 sections, including shaved oral and aboral borders).  Expanding, effacing and replacing the inner muscular layer is a non-encapsulated, mildly infiltrative, multilobular, densely cellular neoplasm arranged in loose bundles and streams, supported by a moderate fibrovascular matrix.  Neoplastic cells are large, spindloid, moderately pleomorphic, with indistinct cell borders, scant to moderate eosinophilic cytoplasm, which contains generally a single oval nucleus, with 1-2 nucleoli and stippled chromatin.  There is moderate anisocytosis and anisokaryosis, with up to 58 mitoses observed within 10 high power fields.  Neoplastic cells multifocally contain up to 6 nuclei.  Where ulcerated, the mucosal surface is overlain by a coagulum of necrotic debris, haemorrhage, with infiltration by moderate numbers of viable and degenerate neutrophils.  Neoplastic cells are not identified at the shaved oral and aboral borders and are not identified within the supporting mesentery.
**Immunohistochemical Description:**
1) CD117: neoplastic cells do not label with this marker.
2) S100: neoplastic cells do not label with this marker.
3) Desmin: neoplastic cells do not label with this marker.
4) Smooth muscle actin: less than 10% of cells throughout the neoplasm exhibit strong intracytoplasmic, granular labelling.
5) Vimentin: Neoplastic cells exhibit strong fine intracytoplasmic labelling.
6) Cytokeratin: Neoplastic cells do not label with this marker.
**Final Diagnosis:**
Compatible with poorly differentiated leiomyosarcoma, high grade, duodenum.
[Soft tissue sarcoma, high grade, duodenum.]
**First Comment:**
Effacing the small intestinal wall, is a spindle cell neoplasm which, given its location and appearance is most likely to be consistent with the presence of either a leiomyosarcoma or gastrointestinal stromal tumour (GIST). Gastrointestinal stromal tumours are thought to be more aggressive, with an increased potential for localised metastasis. These two entities cannot be differentiated using light microscopy alone, immunohistochemistry is recommended for their differentiation (4 markers) and is available on request. Given the high mitotic index and moderate pleomorphism, I would be suspicious that this tumour will display a more aggressive behaviour. The neoplasm appears removed from the planes of tissue examined, however monitoring of this patient is warranted.
Addendum 1:
The initial panel of immunohistochemical labels are somewhat non-specific. There is an absence of CD117 labelling which renders a diagnosis of gastrointestinal stromal tumour unlikely. The muscle filament labels are also negative or scant, therefore the low number of cells that label with smooth muscle actin makes the diagnosis of leiomyosarcoma uncertain. A further panel of cytokeratin and vimentin will be performed and reported when available. I would like to exclude the presence of a poorly differentiated carcinoma and confirm the labelling pattern for vimentin: the original histologic diagnosis therefore remains appropriate until further labels are available.
Addendum 2:
Further immunohistochemical labelling confirms the presence of a mesenchymal neoplasm and excludes the possibility of a poorly differentiated carcinoma. The cell of origin remains uncertain because the neoplastic population is poorly differentiated however a smooth muscle origin (leiomyosarcoma) may be suspected given the scant labelling for smooth muscle actin. I have altered the diagnosis to reflect this. The behaviour remains less predictable but, as perviously described, there is a high mitotic index and pleomorphism therefore an aggressive clinical course might be anticipated. Metastasis from this site cannot be excluded.

Case 5

**Clinical History:**
Duodenal mass.
**Macroscopic Description:**
A beige section of tubular tissue measuring  70x45x40mm, with a mass measuring  40x30mm. Representative sections taken.
**Microscopic Description:**
Duodenum (3 sections). Widely infiltrating the tunica muscularis is a moderately well demarcated, partially encapsulated, densely cellular neoplasm. Neoplastic cells form short haphazard interlacing bundles which are supported by small amounts of stroma. Individual neoplastic cells are spindle-shaped, have indistinct cell borders and contain moderate amounts of eosinophilic fibrillar cytoplasm. Nuclei are oval, contain 1-3 small distinct nucleoli and finely stippled chromatin. 5 mitotic figures were identified within 10 HPF and there is a mild degree of anisocytosis and anisokaryosis. Frequently, the neoplastic cells are replaced by coalescing aggregates of eosinophilic granular material admixed with karyorrhectic debris (necrosis). Infiltrating the neoplastic stroma are moderate numbers of lymphocytes, plasma cells and neutrophils. Occasional discrete lymphoid aggregates are present throughout. Moderate numbers of lymphocytes and plasma cells multifocally infiltrate the adjacent lamina propria. There are no neoplastic cells identified within the shaved soft tissue margins.
**Immunohistochemical Description:**
90% of neoplastic cells exhibit moderate cytoplasmic expression of CD117.
Neoplastic cells do not express smooth muscle actin, desmin or vimentin.
There is patchy staining of the neoplastic population with vimentin (background staining).
**Final Diagnosis:**
Gastrointestinal stromal cell tumour, duodenum.
First Comment:
This mass represents a sarcoma for which differential diagnoses include a leiomyosarcoma  gastrointestinal stromal cell tumour or a fibrosarcoma.  Immunohistochemistry may help to further characterise this population; please contact us if you would like to order this test.  This tumour has been fully excised in the specimen examined.
Addendum:
The immunohistochemical staining profile of the neoplastic cells indicates that this tumour is a gastrointestinal stromal tumour (GIST) rather than a leiomyosarcoma or other soft tissue sarcoma type. The neoplastic cells exhibit strong expression of CD117 and this  common feature of GISTs is thought to reflect constitutive activity of the receptor following neoplastic transformation. CD117 is the target for the recently introduced receptor tyrosine kinase antagonists (eg: toceranib (Palladia), Zoetis). Therefore whilst these drugs are marketed towards treatment of mast cell tumours, they would also be expected to have some activity towards GISTs. You may wish to discuss these results with an oncologist to obtain advice on how best to proceed with any further treatment of this dog.

Case 6

**Clinical History:**

Jejunum, liver L lobe mass, mesenteric LN. Vomiting, mid jejunal structure/ mass- resect. Also biopsy of jejunal/ mesenteric LN. Liver ? red/ cream raised nodules, biopsy of a large ?.

**Gross description:**

A- Jejunum

A beige grey section of tubular tissue measuring 70x40x25mm. Representative sections taken.

B- Liver biopsy

A beige-brown irregular wedge of tissue measuring 15x12x10mm. Bisected. All tissue used.

C- Mesenteric lymph node

A beige irregular wedge of tissue measuring 4x2x2mm. Embedded whole. All tissue used.

**Histological description:**

A. Jejunum (4 sections). Widely infiltrating the submucosa and intestinal muscular wall is a poorly demarcated, unencapsulated and moderately cellular neoplastic mass. Neoplastic cells form irregular and often tortuous tubular structures and small nests. The tubules are often dilated and filled with mucoid material. Individual neoplastic cells are polygonal with indistinct cell borders and small to moderate amounts of eosinophilic cytoplasm. Nuclei are round to oval with vesiculated chromatin and mostly 1 distinct small to medium sized magenta nucleolus. Mitoses are 41 in 10 high power fields and sometimes bizarre. Occasional apoptotic cells are interspersed. There is marked anisocytosis and anisokaryosis. Often, neoplastic cells are surrounded by moderate amounts of fibrovascular tissue (desmoplasia). The neoplastic cell

population multifocally spreads into the mesenteric adipose tissue. In the examined sections, neoplastic cells appear fully excised. In sections from the proximal and distal surgeon-cut edge, no evidence for the presence of neoplastic cells is observed.

B. Liver (2 sections). The overall hepatic tissue architecture is retained. In multifocal to confluent areas, the hepatic parenchyma is expanded and the adjacent parenchyma is slightly compressed. In these areas, the spacing of portal tracts and central veins is increased. Portal tracts are however still evident. In these areas, individual hepatocytes are decreased in size. They contain markedly vacuolated cytoplasm. The cytoplasm contains variably sized but generally small vacuoles containing clear space. Nuclei are round to oval with stippled chromatin

and inconspicuous nucleoli. Mitoses are less than 1 in 10 high power fields. In adjacent areas, the hepatocytes often contain moderate amounts of yellow-green cytoplasmic material (bile or lipofuscin).

C. Mesenteric lymph node (1 section). This is a slightly fragmented section of mesenteric lymph node. Generally, the tissue architecture is retained. The section represents cortex and medullary cords, which contain abundant small, inconspicuous lymphocytes. Multifocal small accumulations of mast cells are present.

**Histological diagnosis:**

A. Adenocarcinoma, jejunum.

B. Nodular hyperplasia, multifocal, marked, liver.

C. Mastocytosis, multifocal, mild, mesenteric lymph node.

**Comment:**

In the submitted sample from the jejunum, an adenocarcinoma is widely infiltrating the submucosa, muscular wall and mesenteric adipose tissue. The neoplastic tissue appears fully excised in the examined sections with no neoplastic cells present in sections from the proximal and distal surgeon-cut edge. However, this may not prove curative, as this tumour would be expected to have potential for transcoelomic spread and metastasis to regional lymph nodes and other organs. Nevertheless, in the submitted mesenteric lymph node there is no evidence for the presence of neoplastic cells. Occasional small clusters of very well differentiated mast cells are present and are considered an incidental finding. In the submitted sample of liver, quite marked nodular areas of hepatocellular regeneration are present. I have considered a hepatocellular tumour, however, the overall lobular architecture is retained. Rather, this may represent a regenerative response to a previous noxious event.

Case 7

**Clinical History:**
Small intestinal mass, intussusception.
**Macroscopic Description:**
A beige grey section of tubular tissue measuring  65x35x25mm. Margins are inked on receipt of tissue. Representative sections taken.
**Microscopic Description:**
Small intestine (3 sections, including oral and aboral borders). Extending from the muscular layer, elevating the overlying mucosa, forming a multi lobular mass that sub totally includes the intestinal lumen, is a well demarcated, unencapsulated, moderately cellular neoplasm arranged in loose bundles and streams, supported by scant fibrous connective tissue. Neoplastic cells are large, fusiform, with indistinct cell borders, abundant eosinophilic cytoplasm that contains a single fusiform nucleus. Nuclei contain indistinct nucleoli and vesicular chromatin. There is mild anisocytosis and anisokaryosis, with 7 mitoses identified in 10 high power fields. The neoplastic population is infiltrated by very low numbers of lymphocytes, plasma cells and occasional neutrophils. Clusters of haemosiderophages are present within the superficial neoplasm subjacent to the overlying mucosa. Multifocally and extensively the mucosal surface is ulcerated, replaced by a  dense coagulum composed of necrotic debris, scant haemorrhage and moderate numbers of degenerate neutrophils. The neoplastic population is not present within the oral and aboral surgeon-cut tissue borders.
**Immunohistochemical Description:**
1) Vimentin: the neoplastic population exhibits diffuse, strong, fine to punctate intracytoplasmic labelling.
2) S100: the neoplastic population exhibits diffuse, scant to moderate, fine intracytoplasmic labelling for this marker.
3) CD117: the neoplastic population does not exhibit labelling for this marker.
4) SMA: the neoplastic population exhibits diffuse, strong, fine intracytoplasmic labelling for this marker.

**Final Diagnosis:**
Leiomyosarcoma, low grade, small intestine.
**First Comment:**
Present within the submitted sections of small intestine there is a tumour originating from the muscular layers of the intestine, forming a pedunculated mass that sub totally occludes the intestinal lumen. An intussusception was not identified grossly although may certainly have been present at surgery, with subsequent reduction prior to our examination. The described intestinal mass is likely to have contributed to inappropriate intestinal motility and formation of the intussusception described clinically. 
The mass is composed of a spindle cell population, with an elevated mitotic index that is not compatible with the presence of a benign tumour such as a leiomyoma. The two primary differential diagnoses are a gastrointestinal stroma tumour and leiomyosarcoma; differentiation of which can be attempted via immunohistochemistry (4 markers) and may have relevance for treatment modality. The neoplasm appears removed from the oral and aboral surgeon-cut tissue borders, indicating full excision of the tumour.
Addendum:
The immunohistochemical labelling pattern is compatible with a smooth muscle origin, the neoplastic population being positive for vimentin, smooth muscle actin and S100. The neoplastic population does not exhibit labelling for CD117 and therefore a gastrointestinal stromal tumour (GIST) is less likely: tyrosine kinase inhibitors are therefore not indicated. This tumour is likely to be locally infiltrative, with lesser propensity for metastasis; the full excision may prove curative.

Case 8

**Clinical History:**

Large pot - abdo intestinal mass. Small pot - Biopsy from pylorus. Blood loss, low iron, scoped stomach NAD except odd looking pylorus, exlap - intestinal mass.

**Gross description:**

A beige-brown section of tubular tissue measuring 60x30x35mm, with a mass measuring 15x15mm. Representative sections taken.

B- 1x beige piece of tissue measuring up to 2mm diameter. Embedded whole. All tissue used.

**Histological description:**

A. Small intestine (3 sections). Arising within the tunica muscularis there is a non-encapsulated neoplasm which protrudes into the intestinal lumen markedly elevating the overlying ulcerated mucosa. Neoplastic cells are arranged in streams and bundles supported by a stoma of well organised blood vessels. Neoplastic cells are spindle shaped with a moderate amount to abundant bright eosinophilic cytoplasm and indistinct cell borders. Nuclei are oval with finely

stippled chromatin and 1 nucleolus. There is moderate anisocytosis and anisokaryosis. 13 mitoses are observed within 10 high power fields. Within the mass there are multiple large areas of necrosis and aggregates of neutrophils. No obvious neoplastic tissue is observed within the surgeon-cut tissue borders of the submitted intestinal segment.

B. Stomach (2 sections). The mucosa of the pylorus is lined by continuous tall columnar epithelium. Glandular tissue is closely packed and separated by narrow fibrous stroma. Intraepithelial lymphocytes are fewer than 1-2 per stretch of 50 epithelial cells. Within the lamina propria numbers of lymphocytes and plasma cells are mildly increased (20-30 per x 40 field). Eosinophils are 1-2 per x 40 field.

**Histological diagnosis:**

A. Sarcoma, small intestine.

B. Gastritis, lymphocytic and plasmacytic, multifocal, mild, stomach.

**Comment:**

The mass within the small intestine is a spindle cell neoplasm. The histological features suggest a leiomyosarcoma; however immunohistochemistry would be required to rule out a gastrointestinal stromal tumour (GIST). This can be performed on this sample upon request (4 antibodies). No obvious neoplastic tissue is observed within the margins of the submitted intestinal segment and therefore complete excision appears to have been achieved. There may

be some potential for metastasis and continued monitoring is recommended.

The pyloric biopsy reveals mild chronic inflammation but there is no evidence of ulceration or neoplasia within the pyloric sample.

Case 9

**Clinical History:**

LN biopsy. Mid jejunal section. Intermittent vomiting and diarrhoea. Intestinal mass, enlarged LN, no metastases found.

**Gross description:**

A: A beige-brown section of tubular tissue measuring 105x25x20mm. Representative sections taken.

B: Lymph node. A beige-brown piece of ovoid shaped tissue measuring 12x12x4mm. Bisected. All tissue used.

**Histological description:**

A. Intestine (5 sections). Extending from the mucosal surface, transmurally expanding the submucosa, muscular layers and bluntly infiltrating the serosa, is a moderately well demarcated, unencapsulated, densely cellular, highly infiltrative neoplasm arranged in sheets and thin cords, supported by the pre-existing stroma. Neoplastic cells are large, round, with variably distinct cell

borders, moderate eosinophilic cytoplasm, which contains a single round nucleus. Nuclei are greater than 2x the size of adjacent erythrocytes, contain indistinct to 1 small nucleolus and coarsely stippled chromatin. There is mild to moderate anisocytosis and anisokaryosis, with up to 11 mitoses identified in a single high power field. Scattered throughout the neoplastic population there are low to moderate numbers of eosinophils, very low numbers of plasma cells and rare neutrophils. The neoplastic population is present within the shaved (oral/aboral) surgeon-cut tissue borders.

B. Lymph node (2 sections). These sections are composed of transverse profiles of lymph node, supported by expected amounts of fibrous connective tissue and bordered by a thin rim of adipose connective tissue. Infiltrating, effacing and subtotally replacing the described lymph node, are sheets of a neoplastic population as described above. The neoplastic population is large, round, with variably distinct cell borders, moderate eosinophilic cytoplasm, which contains

a single round nucleus. Nuclei measure greater than 2x the size of adjacent erythrocytes, contain indistinct to 1 small nucleolus and stippled chromatin. There is mild to moderate anisocytosis and anisokaryosis, with up to 17 mitoses identified in a single high power field. Scattered throughout the neoplastic population there are low numbers of eosinophils and rare plasma cells. The medullary sinuses contain moderate numbers of extravasated erythrocytes

and low numbers of haemosiderophages.

**Histological diagnosis:**

A. Compatible with null cell lymphoma, large cell, high grade, intestine.

B. Compatible with null cell lymphoma, large cell, high grade, lymph node.

**Comment:**

The mass within the intestine is composed of a round cell tumour that is also present effacing the submitted lymph node. This is a highly infiltrative tumour, which extends to the oral/aboral borders of the intestine. The neoplastic population is large, round, with nuclei greater than 2x the size of adjacent erythrocytes, compatible with a large cell designation. The mitotic index within

both the intestine and lymph node is greater than 10 mitoses per single high power field, which is compatible with a high histologic grade. There are low to moderate numbers of eosinophils scattered throughout the neoplastic population, therefore I did consider if this might represent a

mast cell tumour, however the cellular morphology is more compatible with that of lymphoma. To confirm this diagnosis, immunophenotype the population and exclude the presence of a mast cell tumour, immunohistochemistry is required. Four markers are required initially and are available on request. Given the involvement of the submitted lymph node, the neoplasm may be expected to involve multiple further draining lymph nodes and/or internal organs. Close

monitoring of this patient is recommended.

Case 10

**Clinical History:**
Intestinal mass resection. Anaemia, suspected GI bleeding, intestinal mass identified on ultrasound. Ex lap. Mass excised with at least 5cm margins. Previous FNAs submitted.
**Macroscopic Description:**
A grey section of tubular tissue measuring 120x65x20mm, with a mass measuring 30x25mm. Representative sections taken.
**Microscopic Description:**
Small intestine (4 sections). Arising within the tunica muscularis there is a moderately well circumscribed, non-encapsulated neoplasm. Neoplastic cells are arranged in broad streams and interlacing bundles, supported by a fine fibrovascular stroma. Neoplastic cells are spindle shaped with abundant bright eosinophilic fibrillar cytoplasm and indistinct cell borders. Nuclei are oval to elongated with finely stippled chromatin and 1 nucleolus. There is mild to moderate anisocytosis and anisokaryosis. 6 mitoses are observed in 10 high power fields. The overlying intestinal mucosa is focally extensively ulcerated. In this area a small cleft extends into the underlying mass. This cleft is bordered by large aggregates of neutrophils admixed with macrophages. In this area there is formation of granulation tissue with numerous capillaries, orientated perpendicular to the cleft. In the adjacent mucosa villi are mildly to moderately blunted. Numbers of lymphocytes and plasma cells in the lamina propria are mildly increased. No obvious neoplastic tissue is identified within sections through the surgeon-cut tissue borders of the submitted intestinal segment.
**Immunohistochemical Description:**
Neoplastic cells exhibit strong cytoplasmic labelling for smooth muscle actin and desmin and weak labelling for vimentin. No labelling for CD117 is identified within neoplastic cells.
**Final Diagnosis:**
Leiomyosarcoma, well differentiated, small intestine.
First Comment:
The mass within the intestinal wall is a spindle cell neoplasm. The histological features suggest a well differentiated leiomyosarcoma. The mass appears well circumscribed and a leiomyoma was considered. However there are occasional mitotic figures and therefore I favour a well differentiated leiomyosarcoma. A gastrointestinal stromal tumour is considered less likely, but can be further ruled out by using immunohistochemistry (4 antibodies). There is focal ulceration of the overlying mucosa and a small foreign body was identified entrapped within the mass. This likely occurred secondary to partial obstruction of the intestinal passage by the tumour. No obvious neoplastic tissue is identified within the surgeon-cut tissue borders of the intestinal segment and complete excision appears to have been achieved. Metastasis of this tumour is less likely, but continued monitoring of the patient is nevertheless recommended.
Addendum:
The neoplastic cells exhibit expression of smooth muscle actin and desmin consistent with a smooth muscle origin and the features are consistent with a leiomyosarcoma. The neoplastic cells are negative for CD117(kit) and this rules out a gastrointestinal stroma tumour.

Case 11

**Clinical History:**
Ex lap following previous results (see cytology). Confirmed thickening of small intestinal wall in one discrete area, omentum adhered, pus present in thickening. R enterectomy performed.
**Macroscopic Description:**
A beige grey section of tubular tissue measuring 60x30x30mm with a sessile mass measuring 25x30mm. Margins are inked on receipt of tissue. Representative sections taken.
**Microscopic Description:**
Small intestine (4 sections). Present at the site of the mass identified grossly, is a locally infiltrative, non-encapsulated, moderately densely cellular neoplastic mass. Neoplastic cells extend from the ulcerated lumen of the intestinal segment and through all muscle layers and into the adjacent mesentery. Neoplastic cells are arranged in densely packed short interlacing streams and bundles, supported by small amounts of loose collagenous matrix. Individual neoplastic cells are spindle shaped, have indistinct cell borders and contain moderate amounts of eosinophilic cytoplasm. Nuclei are oval to elongate oval and contain stippled to dispersed chromatin with 1-2 moderately prominent, moderately large nucleoli. There is a moderate degree of anisokaryosis and anisocytosis throughout the neoplastic cell population. Mitoses are 18 per 10 high power fields. Extensive areas of central necrosis are present within the mass amongst which are large numbers of degenerate neutrophils. No neoplastic cells are apparent at the surgeon-cut ends of the intestinal segment.
**Immunohistochemical Description:**
Neoplastic cells do not stain for smooth muscle actin, desmin, S100 or CD117.
Surrounding smooth muscle cells exhibit moderate to intense positive cytoplasmic immunohistochemical staining for smooth muscle actin and desmin. Scattered well differentiated mast cells within the intestinal wall and mesentery exhibit moderate membranous immunohistochemical staining for CD117.
**Final Diagnosis:**
Fibrosarcoma, high grade, small intestine.
First Comment:
As suspected, this mass represents a sarcoma with the differential diagnosis being a leiomyosarcoma and a gastrointestinal stromal tumour. Distinguishing between these tumour types would require immunohistochemistry and this can be performed on request (4 antibodies). Distinguishing between these tumour types may have clinical benefits, as a gastrointestinal stromal tumour would be expected to express KIT, which is the target for the recent generation of receptor tyrosine kinase antagonists. In contrast, a leiomyosarcoma would not be expected to express KIT, and therefore would be much less likely to respond to these drugs. The mass does appear to have been fully excised with no neoplastic cells at the ends of the tissue segment, although there would be potential for metastasis from this site.
Addendum:
The immunohistochemical staining profile of the neoplastic cells indicates that it is not a leiomyosarcoma as the neoplastic cells do not stain with either of the smooth muscle markers, smooth muscle actin or desmin. Additionally, the neoplastic cells do not exhibit positive staining for Kit (CD117) and therefore this mass is not a gastrointestinal stromal tumour. The neoplastic cells do not stain for S100 and therefore this mass is also not a peripheral nerve sheath tumour.
Therefore, given this immunohistochemical staining profile and the histological features, the mass is most likely a fibrosarcoma, as these tumours would not be expected to express any of the immunohistochemical stains used in this panel. 
The mass would still have some potential for metastasis from this site, but based on the absence of positive staining for Kit, it may not respond to receptor tyrosine kinase antagonists.

Case 12

**Clinical History:**

Intestinal stricture at jejuno ileal junction. 3 week history anorexia and vomiting.

**Gross description:**

A beige-brown section of tubular tissue measuring 40x37x30mm. Representative sections taken.

**Histological description:**

Jejuno-ileal junction (7 sections). In the centre of the submitted segment of intestine with the grossly noted stricture, there is a neoplastic mass arising from the mucosal layer. The neoplastic cells partially efface the mucosa and extend through the submucosa, muscularis, serosa and into the attached mesenteric adipose. The neoplasm is composed of individual and small clusters of

polygonal neoplastic cells. The cells have distinct cell borders and contain a moderate to abundant amount of granular cytoplasm that ranges from eosinophilic to amphophilic. The abundant cytoplasm often slightly compresses the nucleus to the periphery. Nuclei are oval with stippled to clumped chromatin and often have 1-3 prominent nucleoli. There is moderate anisocytosis and anisokaryosis, and there are up to 5 mitotic figures in a single high power field.

Occasional aggregates of neoplastic cells produce abundant mucoid exudate and neoplastic cells within the mesentery are surrounded by fibrosis. Throughout the mesentery and extending slightly within the wall of the jejunum, there is a moderate, perivascular lymphocytic infiltrate. Sections of tissue from the margins of the submitted intestinal segment show a mild lymphocytic

and plasmacytic infiltrate within the mucosa. There is no evidence of neoplastic cells in these marginal sections.

**Histological diagnosis:**

Carcinoma, mucinous-type, jejuno-ileal junction.

**Comment:**

The submitted segment of intestine shows a markedly infiltrative malignant neoplasm consistent with a mucinous carcinoma that extends transmurally as well as into the attached mesentery. These neoplasms are expected to behave in both a locally infiltrative manner and have a relatively high potential for metastasis via the lymphatic channels. Given this feature, further monitoring of the draining lymph nodes would be warranted even though the entire segment of affected intestine appears to have been resected.

Case 13

**Clinical History:**

1 - Jejunal obstruction next to abnormal jejunum with thickened wall/stenosis ?tumour. Ends inked. Cut on anti-mesenteric border.

2 - Elevated liver enzymes.

**Gross description:**

A- Jejunum. A beige section of tubular tissue measuring 130x25x20mm. Representative sections taken.

B- Liver. A brown irregular wedge of tissue measuring 12x4x4mm. Bisected. All tissue used.

C- Liver. A brown irregular wedge of tissue measuring 15x6x5mm. Bisected. All tissue used.

**Histological description:**

A. Jejunum (3 sections). Extending from the mucosa into the submucosa and smooth muscular wall and multifocally infiltrating the adjacent mesentery, is a poorly demarcated, poorly cellular neoplastic mass. Neoplastic cells form multiple small nests and clusters, irregular tubular structures or infiltrate as individual cells. They are often surrounded by large amounts of fibrous

connective tissue (desmoplasia). Neoplastic tubules are often dilated and filled with abundant pale basophilic mucous material. Nests of neoplastic cells are often surrounded by lake-like accumulations of similar material. Individual neoplastic cells are polygonal with indistinct cell borders and moderate amounts of amphiphilic cytoplasm. Nuclei are round to oval with coarsely stippled chromatin and one to several small to medium-sized magenta nucleoli. Mitoses are 2 in 10 HPF. There is marked anisocytosis and anisokaryosis. The mucosa is multifocally ulcerated. In sections from the proximal and distal surgeon-cut edge, there is no evidence for the presence of neoplastic cells.

B. Liver (4 sections). Portal tracts and central veins are evenly spaced in these liver biopsies. Portal tracts are mildly infiltrated with lymphocytes, plasma cells and neutrophils. Occasionally, portal tracts are mildly expanded by fibrosis. Multifocal biliary hyperplasia is observed. Occasionally, hepatocytes at the portal-parenchymal interface are replaced by fibrosis and thin strands of fibrous connective tissue occasionally extend a short way into the parenchyma. Occasionally, individual hepatocytes in the parenchyma are replaced by accumulations of Kupffer cells and macrophages (microgranulomas). Mildly increased numbers of neutrophils are present in the sinusoids.

**Histological diagnosis:**

A. Adenocarcinoma, jejunum.

B. Hepatitis, unspecific-reactive, lymphocytes, plasma cells, neutrophils with bile duct hyperplasia, multifocal, mild and fibrosis, portal, multifocal, minimal.

**Comment:**

As suspected, a carcinoma is widely infiltrating from the mucosa into the submucosa, muscular wall and mesentery in the submitted sample of jejunum.

This tumour would be expected to behave in a locally aggressive manner with potential for metastasis from the site. In sections from the inked proximal and distal surgeon-cut edge, there is no evidence for the presence of neoplastic cells, suggesting complete local excision has been achieved.

In the liver, a mild unspecific reactive hepatitis is observed and likely related to inflammation in the intestine caused by the neoplasia. Occasional microgranulomas are observed and may reflect mild bacterial embolic showering from the intestine. Occasionally, the portal-parenchymal

interface is slightly ragged due to minimal fibrosis extending occasionally into the parenchyma. I have considered ‘chronic hepatitis’ as differential diagnosis. However, typical interface-type hepatitis with hepatocellular apoptosis is not a feature in these samples and therefore, this is unlikely.

Case 14

**Clinical History:**
Duodenal mass.  Biopsy suggestive of stromal tumour.
**Macroscopic Description:**
Duodenal mass. A beige-brown section of tubular tissue measuring 55x20x15mm. Representative sections taken. Tissue fixed further prior to processing.
**Microscopic Description:**
Duodenum (4 sections).   Arising at the base of the mucosa near the level of the muscularis mucosa, there is a poorly demarcated, infiltrative, neoplastic mass that extends through the submucosa and nearly through the muscularis.  The mass is composed of densely cellular, short, intersecting streams and bundles of spindle cells supported by a scant fibrovascular stroma.  Individual neoplastic spindle cells have indistinct cell borders and contain a scant to occasionally moderate amount of eosinophilic cytoplasm.  Nuclei are oval to elongate with stippled chromatin and indistinct nucleoli.  There is mild anisocytosis and anisokaryosis, and no mitotic figures are seen in 10 high power fields.  At the centre of the mass is a poorly demarcated region with well vascularised stromal tissue and multifocal aggregates of macrophages.  Occasionally these aggregates of macrophages centre around fragment of foreign material that ranges from small, angular, translucent to basophilic fragments to angular, slightly pigmented fragments of tissue that may be consistent with hair shafts.  There are also occasional multinucleated giant cells within this region and there are numerous pigment-laden macrophages admixed with a small amount of haemorrhage. Sections of tissue from the orad and aborad margins are within normal limits with no evidence of the neoplastic mass.
**Immunohistochemical Description:**
Approximately 75% of neoplastic cells show weak to moderate, positive, cytoplasmic immunoreactivity for CD117 (c-kit), and nearly 100% of neoplastic cells show weak to moderate positive cytoplasmic immunoreactivity for vimentin.  
Neoplastic cells are negative for smooth muscle actin (SMA) and S100.  Small arterioles show strong positive SMA staining in myofibres (internal positive control) and there are numerous cells within the myenteric plexus that exhibit positive S100 staining (internal positive control).
**Final Diagnosis:**
Sarcoma, with focal granulomatous inflammation and intralesional foreign material, duodenum.
First Comment:
As suspected, there is a mass within the wall of the submitted segment of duodenum.  Histologic features are consistent with a sarcoma and differential diagnoses include either a gastrointestinal stromal tumour (GIST) or a low grade leiomyosarcoma.  Additional immunohistochemical staining would be recommended to further characterise the neoplastic cells and differentiate between these two neoplasms.  In either case, this mass would be expected to be locally infiltrative and may have some potential for metastasis from this site (more likely with a GIST).  The mass does appear to have been fully excised and the sections of tissue from the margins of the submitted segments are within normal limits.  There is an interesting focus in the centre of the neoplastic mass with a few aggregates of granulomatous inflammation that is surrounding fragments of foreign material.  I suspect this foreign material originated from the lumen of the duodenum and has been transplanted through the injured part of the wall due to the neoplasm.  Again, both the neoplasm and the area of granulomatous inflammation appear to have been resected in entirety.  Please call if you would like to request additional immunohistochemical stains for the neoplasm (panel of 4 antibodies).
Addendum:
Immunohistochemical staining indicates that the neoplastic cells are positive for vimentin, consistent with a mesenchymal origin, and they exhibit positive staining for CD117 (c-kit), indicative of a gastrointestinal stromal tumour (GIST).  Negative SMA staining helps to rule out a leiomyosarcoma. GISTs are expected to behave in a locally infiltrative manner and have some potential for metastatic disease.  In this case, the mass does appear to have been fully excised, thus decreasing the likelihood for local recurrence.

Case 15

**Clinical History:**

V+ D+

**Gross description:**

A: Duodenal mass + intestinal ??. A beige grey section of tubular tissue measuring 160x45x35mm with a thickened area measuring 80x40mm. An intraluminal aggregate of hard material approximately 20mm in length found.

Representative sections taken including shave proximal and distal margins.

B: Mesenteric LN x2. 2 beige grey piece of ovoid shaped tissue measuring up to 25x20x10mm. Bisected. All tissue used.

**Histological description:**

A. Small intestine (7 sections). At the site of the thickened area identified grossly, the mucosal epithelium has undergone segmental neoplastic transformation with neoplastic cells extending throughout all layers of the intestinal wall and into the adjacent mesentery. Neoplastic cells are arranged in nests, cords and tubular structures, supported by large amounts of fibrovascular connective tissue stroma and multifocally neoplastic cells are surrounded by clusters of

mucinous material. Individual neoplastic cells are polygonal to cuboidal and contain moderate to large amounts of eosinophilic cytoplasm with indistinct cell borders. Nuclei are moderately large, oval to round and contain stippled to dispersed chromatin with 1-2 large prominent nucleoli. Mitoses within this cell population are 12 per 10 high power fields. There is a moderate degree of

anisokaryosis and anisocytosis throughout the neoplastic cell population. Extensive accumulations of lymphocytes , neutrophils and lesser numbers of plasma cells are infiltrating around the neoplastic cells within the mesentery. Neoplastic cells are widely infiltrating into an adjacent lymph node within the mesentery. The mass does not extend to the proximal or distal surgeon-cut edges of the sample.

B. Mesenteric lymph node (2 sections). There is marked lymphoid follicular hyperplasia throughout the lymph nodes. Multiple clusters of neoplastic cells are present within the subcapsular sinuses and extend into the adjacent lymph node parenchyma. One of the lymph nodes is much more severally affected than the other.

**Histological diagnosis:**

Carcinoma, transmural with lymph node metastasis, mesentery and small intestine.

**Comment:**

There is a highly infiltrative carcinoma that has developed from within the intestinal mucosa which has infiltrated throughout all layers of the intestinal wall and has metastasised to two of the associated mesenteric lymph nodes. Therefore there would be significant potential for further metastasis from these sites. The foreign body identified grossly within the intestinal segment may have contributed to the clinical signs, but would likely have lodged at this site following partial obstruction of the lumen by the neoplastic cell infiltrates.

Case 16

**Clinical History:**

Intestinal blood loss and change in appetite. No evidence of metastatic spread.

**Gross description:**

A beige grey section of tubular tissue measuring 80x40x30mm. Representative sections taken.

**Histological description:**

Jejunum (4 sections). Disrupting the muscularis layers and extending to the ulcerated mucosal surface within the intestinal segment at the site of the mass identified grossly, is a moderately well demarcated, broadly infiltrative, non encapsulated neoplastic mass. The mass has been fully excised with no neoplastic cells at the proximal or distal surgeon-cut edge of the sample.

Neoplastic cells are arranged in intersecting bundles and long streams, supported by small amounts of fibrovascular connective tissue stroma. Individual neoplastic cells are spindle shaped to strap-like and contain abundant eosinophilic cytoplasm with indistinct cell borders. Nuclei are elongate oval, moderately large and contain dispersed stippled chromatin with 1-2 small nucleoli. Mitoses are 4 per 10 high power fields. There is a moderate degree of anisokaryosis throughout the neoplastic cell population. Occasional areas of confluent necrosis are present within the mass, representing less than 10% of the total tissue area.

**Histological diagnosis:**

Leiomyosarcoma, low grade, jejunum.

**Comment:**

This mass represents a tumour of smooth muscle origin. The presence of occasional mitoses amongst the neoplastic cell population and the low degree of invasion into the surrounding muscularis layers would be indicative of a leiomyosarcoma, rather than an entirely benign leiomyoma. However, given the well-differentiated nature of the neoplastic cells, despite this histological diagnosis, surgical excision may still prove curative and has been achieved.

Case 17

**Clinical History:**

Weight loss, melaena, lethargy, anaemia. Mid jejunal mass on u/s, staging negative. Exlap: distal jejunal mass removed with approximately 10cm margins and end to end anatomises. NAD.

**Gross description:**

A beige section of tubular tissue measuring 90x40x25mm, with a mass measuring 25x20mm. Representative sections taken.

**Histological description:**

Jejunum (3 sections). Within the submucosa and tunica muscularis, there is a non-encapsulated neoplasm. Neoplastic cells are arranged in streams and bundles supported by a fine fibrovascular stroma. Neoplastic cells are spindle shaped to elongated with a moderate amount to abundant bright eosinophilic fibrillar cytoplasm and indistinct cell borders. Nuclei are oval with finely stippled chromatin and one nucleolus. There is mild to moderate anisocytosis and anisokaryosis. Two mitoses are observed in 10 high power fields. The overlying mucosa is, in some areas, ulcerated and aggregates of neutrophils and fibrin are present on the ulcerated surface and within the underlying mass. In other areas, the overlying mucosa is well organised. Within sections through the surgeon-cut tissue borders of the submitted intestinal segment, no obvious neoplastic tissue is observed.

**Histological diagnosis:**

Sarcoma, jejunum.

**Comment:**

The mass within the wall of the jejunum is a spindle cell neoplasm. The histological features suggest a leiomyosarcoma, but a gastrointestinal stromal tumour is a potential differential diagnosis. Immunohistochemistry can be performed on requests for further differentiation of these tumours (4 antibodies). No obvious neoplastic tissue is observed within the surgeon-cut

tissue borders of the submitted sample. Therefore complete excision appears to have been achieved. Given the low mitotic count, metastasis from this site is considered less likely but continued monitoring would nevertheless be prudent.

Case 18

**Clinical History:**

Intestinal mass, mesenteric LN biopsy. Weight loss, vomiting and hypoalbuminaemia. AUS revealed intestinal mass. Ex-lap confirmed mass lesion in proximal jejunum. Also milky serosal lesions in ileum.

**Gross description:**

A: Intestinal mass. A beige grey section of tubular tissue measuring 110x45x30mm with a mass measuring 35x25mm. Representative sections taken.

B: A beige-brown irregular wedge of tissue measuring 7x3x3mm. Embedded whole. All tissue used.

**Histological description:**

A. Proximal jejunum (4 sections). The mucosa, submucosa and tunica muscularis are multifocally infiltrated by a non-encapsulated neoplasm. Neoplastic cells are arranged in sheets, supported by a fine fibrovascular stroma. Neoplastic cells are round with a moderate amount of eosinophilic cytoplasm and indistinct cell borders. Nuclei are round to oval, measure approximately 1.5 times the diameter of adjacent erythrocytes, with finely clumped chromatin and one nucleolus. There is moderate anisocytosis and anisokaryosis. Mitoses average 5-6 per

high power field. Multifocally neoplastic cells extend into the adjacent mesentery. Within sections through the surgeon-cut tissue borders there are areas in which the lamina propria is infiltrated by neoplastic cells, similar to those described above.

B. Mesenteric lymph node (1 section). The lymph node architecture is moderately distorted. Within the cortex there are occasional follicles. These are multifocally moderately compressed by subcapsular and medullary infiltrates of round cells, arranged in sheets. These have similar features to the neoplastic population described above for (A).

**Histological diagnosis:**

A. Round cell tumour, consistent with lymphoma, proximal jejunum.

B. Round cell tumour, consistent with lymphoma, mesenteric lymph node.

**Comment:**

The mass within the proximal jejunum is a round cell tumour. The histological features are cannot be confirmed and there may be local regrowth at the site.

consistent with lymphoma. Other round cell tumours such as mast cell tumour are considered much less likely. Further characterisation of the neoplastic population using immunohistochemistry can be performed on request (lymphoma typing). The mesenteric lymph node biopsy is small but there are similar round cells within the cortex and medulla. These appear to partially compress the follicles and therefore these are likely neoplastic lymphocytes.

Lymph node hyperplasia cannot be fully ruled out but is less likely. This tumour has potential for further metastasis from this site. Neoplastic cells are also present within one of the sections through the surgeon-cut tissue border of the submitted segment. Therefore complete excision cannot be confirmed and there may be local regrowth at the site.

Case 19

**Clinical History:**

Mesenteric lymph node and small intestinal mass.

**Gross description:**

A:A beige-brown section of tubular tissue measuring 100x60x60mm with a portion of a mass measuring 40x60mm. Representative sections taken.

B: Mesenteric lymph node.

A beige-brown irregular wedge of tissue measuring 7x3x3mm. Embedded whole. All tissue used.

**Histological description:**

A. Small intestine (4 sections). The intestinal mucosa is widely effaced by a non-encapsulated neoplasm. Neoplastic cells multifocally extensively infiltrate the underlying submucosa, tunica muscularis and in some area extend to the serosa. Neoplastic cells are arranged in sheets, supported by variable amounts of collagenous stroma. The neoplastic cells are round with a moderate amount of pale eosinophilic cytoplasm and indistinct cell borders. Nuclei are oval, measure approximately 2-2.5x the diameter of adjacent erythrocytes, with finely stippled

chromatin and 1-2 nucleoli. There is moderate anisocytosis and anisokaryosis. 24 mitoses are observed in 10 high power fields. The neoplastic cells are multifocally admixed with large numbers of eosinophils. Within a section through one surgeon-cut tissue border, represented within the submitted sample, no obvious neoplastic tissue is observed.

B. Lymph node (1 section). The section comprises a small fragments of lymph node. Within the cortex there are multiple well organised follicles, which exhibit pale germinal centres. Multifocally the trabeculae are expanded by fibrous tissue. Within medullary cords there are moderate numbers of plasma cells. Multifocally large numbers of eosinophils are present within the medullary sinuses.

**Histological diagnosis:**

A. Round cell tumour, small intestine.

B. Follicular hyperplasia and sinus eosinophilia, multifocal, moderate, lymph node.

**Comment:**

The mass within the small intestine is a round cell tumour. The main differential diagnoses for this tumour are a mast cell tumour or lymphoma. I have requested a Toluidine Blue stain to determine if mast cell granules can be identified in these cells and the results will be reported by under separate cover. Should this prove inconclusive, immunohistochemistry would be useful for further characterisation of the neoplastic population (4 antibodies). Only half of the mass and only one surgeon-cut intestinal margin was received. Within this one margin no obvious neoplastic tissue is identified; however as this is an incomplete sample, full excision cannot be assessed. No obvious neoplastic tissue is observed within the small lymph node biopsy.

Case 20

**Clinical History:**

Chronic blood loss anaemia, proximal duodenal mass removed by enterectomy and biopsies of hepatic and splenic nodules.

**Gross description:**

A: Duodenum (mass). A beige-brown section of tubular tissue measuring 100x35x30mm with a mass measuring 40x30mm. Representative sections including shave margins taken.

B: Hepatic nodule. A brown irregular piece of tissue measuring 4x4x5mm. Bisected. All tissue used.

C: Splenic nodule. A brown irregular wedge of tissue measuring 25x22x25mm with a mass measuring 15x15mm. Representative sections taken.

**Histological description:**

A. Duodenum (5 sections). At the site of the mass identified grossly, the mucosal epithelium has undergone segmental neoplastic transformation. Neoplastic cells extend from the ulcerated submucosal surface into the underlying submucosa and muscularis layers. Neoplastic cells are arranged in nests, cords and occasionally in tubular structures, supported by moderate amounts

of fibrovascular connective tissue stroma. Individual neoplastic cells are polygonal, have variably distinct cell borders and contain moderate amounts of eosinophilic cytoplasm. Nuclei are oval and contain stippled to dispersed chromatin with 1-2 moderately prominent nucleoli. Mitoses are 2-4 per high power field. Multifocally, the neoplastic cells are infiltrating into the subserosal

stroma and mesentery. Occasionally neoplastic cells extend into the lumen of thin-walled vessels. No neoplastic cells are apparent at the proximal or distal surgeon-cut edges of the intestinal segment.

B. Liver (2 sections). The hepatic parenchyma throughout the sections is well organised with generally regularly-spaced central veins and portal triads. There is segmental, moderate to marked cloudy swelling of hepatocytes. Scattered neutrophils, lymphocytes and plasma cells are present within portal triads and around central veins. No neoplastic cells are apparent.

C. Spleen (2 sections). The splenic parenchyma is disrupted by a nodular proliferation of large, well organised lymphoid follicles. Some of the follicles coalesce. Large numbers of plasma cells and scattered Mott cells are present between the follicles. Moderate accumulations of haematopoietic precursors are present within the remaining red pulp.

**Histological diagnosis:**

A. Carcinoma, duodenum.

B. Cloudy swelling, segmental, marked, liver.

C. Nodular lymphoid hyperplasia, focal, marked, spleen.

**Comment:**

The duodenal mass represents a highly invasive carcinoma that has extended throughout all levels of the intestinal wall. The neoplastic cells extend into the adjacent mesentery and in some areas infiltrate into small vessels. There would therefore be significant potential for metastasis from this site.

No neoplastic cells are apparent within the hepatic nodule, but rather this nodular focus likely reflects an area of macronodular hepatocyte hyperplasia.

Similarly, no neoplastic cells are apparent within the splenic mass, but rather this mass represents a benign area of marked lymphoid hyperplasia.

Case 21

**Clinical History:**

Intestinal mass. Liver biopsy.

**Gross description:**

A- Liver. A brown irregular wedge of tissue measuring 20x10x8mm. Bisected. All tissue used.

B- A beige grey section of tubular tissue measuring 185x30x25mm. Representative sections taken.

**Histological description:**

A. Liver (2 sections). The hepatic architecture is preserved. Portal areas are multifocally mildly expanded by mature fibrous tissue and occasional portal areas exhibit multiple profiles of small bile ductules. Infiltrating the portal areas there are low numbers of lymphocytes and plasma cells. Hepatocytes within periacinar and mid-zonal areas are multifocally mildly swollen and have finely

vacuolated cytoplasm.

B. Small intestine (6 sections). Arising from the intestinal mucosa and multifocally infiltrating the underlying submucosa and tunica muscularis, there is a non-encapsulated neoplasm. Neoplastic cells are arranged in cords and form tubular structures, supported by a fibrous stroma. Neoplastic cells are polygonal with a moderate amount of eosinophilic cytoplasm and indistinct cell borders. Nuclei are oval with finely clumped chromatin and 1-2 magenta nucleoli. There is

moderate anisocytosis and anisokaryosis. 21 mitoses are observed in 10 high power fields. Neoplastic cells multifocally surround aggregates of pale basophilic mucinous material. Occasional neoplastic cells exhibit large accumulations of pale basophilic mucinous material within the cytoplasm. No obvious neoplastic tissue is observed within sections through the surgeon-cut tissue borders of the submitted intestinal segment.

**Histological diagnosis:**

A. Hepatocellular cloudy swelling and portal fibrosis, multifocal, mild, liver.

B. Carcinoma, small intestine.

Comment:

The mass within the small intestine is a carcinoma, arising from the intestinal epithelium. This tumour exhibits extensive infiltrative growth into the intestinal wall and extends to the mesentery in some areas. No obvious neoplastic tissue is observed within the sections through the surgeon-cut tissue borders. This tumour would however be expected to have potential for metastasis from this site. No obvious neoplastic tissue is observed within the liver biopsy. The changes within the liver are likely reactive and potentially age-related. No significant inflammation or hepatocellular loss is observed.

Case 22

**Clinical History:**

Progressive anorexia and weight loss with intermittent vomiting. Abdo scan- intestinal mass and enlarged adjacent mesenteric LN. Ex lap- 4.5 cm thickened segment of distal duodenum and enlarged mesenteric LN excised.

**Gross description:**

A beige grey section of tubular tissue measuring 145x75x35mm. Representative sections taken.

**Histological description:**

Duodenum (7 sections). Within the thickened area of duodenum, arising in the mucosal epithelium is a poorly demarcated, highly infiltrative, neoplastic mass that appears fully excised with the resected segment of duodenum. The neoplastic cells are arranged into irregular tubules as well as small clusters or individually, all supported by a moderate fibrovascular stroma. The individual neoplastic cells are polygonal with indistinct borders and contain a moderate amount of eosinophilic cytoplasm. Nuclei are oval to slightly irregular, often with vesicular chromatin and 1-3 variably sized, prominent nucleoli. There is moderate anisocytosis and anisokaryosis, and there are often up to 5 mitotic figures in a single high power field. Numerous neoplastic cells are apoptotic or necrotic, characterised by a shrunken rounded profile with hypereosinophilic

cytoplasm and karyorrhectic nuclei. Many of the neoplastic tubules contain a small amount of mucoid material, or sloughed neoplastic cells or aggregates of macrophages. Neoplastic cells extend through the submucosa, muscularis, serosa and out into the adjacent mesentery. The mesenteric lymph node is completely effaced by the neoplastic population of cells with only scant aggregates of small lymphocytes remaining at the periphery of the tissue. Neoplastic cells also

extend into the perinodal adipose tissue of the surrounding mesentery. Sections of tissue from the labelled proximal and distal ends of the jejunum are free of neoplastic cells. Within these sections, there is a mild infiltrate of plasma cells throughout the lamina propria, and occasional crypts are moderately dilated and filled with eosinophilic debris and pyknotic nuclear debris (crypt abscesses).

**Histological diagnosis:**

Adenocarcinoma, transmural with lymph node metastasis, duodenum and mesenteric lymph node.

**Comment:**

The thickened segment of duodenum is due to a transmural adenocarcinoma. This mass is markedly infiltrative and has extended into the surrounding mesentery and has metastasised to the adjacent lymph node. Although the entire mass appears to have been excised, further metastatic spread is possible. Further monitoring of additional lymph nodes as well as the abdominal cavity for potential carcinomatosis would be warranted.

Case 23

**Clinical History:**

Intestinal mass, chronic history of vomiting (years). Staging negative on abdo ultrasound and 3 thoracic radiographs? Neoplasia (eg. adenocarcinoma, ??). ?Ulcerated inflammatory lesion (IBD and ulcer)

**Gross description:**

A: Liver. A brown irregular wedge of tissue measuring 14x6x7mm. Bisected. All tissue used.

B: Intestinal margins. 8 beige brown irregular pieces of tissue measuring up to 6x2x1mm. Embedded whole. All tissue used.

C: A beige-brown section of tubular tissue measuring 155x30x25mm. Representative sections taken.

**Histological description:**

A. Liver (2 sections). These sections generally retain lobular architecture. Portal tracts contain expected components with occasional minimal mixed inflammatory infiltrates including plasma cells, lymphocytes, and neutrophils. There are frequent, patchy areas of mild hepatocellular degeneration with slight cytoplasmic vacuolation. In many regions, the hepatic cords appear very mildly atrophied with corresponding mild dilation of the sinusoids. Sinusoids often contain scattered neutrophils. There are occasional, variably demarcated, small nodules up to 1 mm in diameter of nodular hyperplasia. Occasionally the hepatocytes within these hyperplastic regions are swollen with lipid vacuolation.

B. Intestinal margins (8 sections). These sections of tissue consist of fragments of mucosa and muscular wall. There is no evidence of a neoplastic mass within these small sections. In the mucosa, the villi appear very slightly blunted, and there is a mild infiltrate of plasma cells and lymphocytes present throughout the lymphocytes.

C. Intestine with mass (4 sections). Representative sections show marked segmental expansion of the intestinal wall consistent with a poorly demarcated neoplastic mass. The mass largely consists of densely packed, long, intersecting streams and bundles of spindle cells supported by a scant amount of fibrovascular stroma. In addition, there are occasional individual or small clusters of large polygonal cells present in between the spindle cells. The spindle cells have indistinct borders with abundant amounts of slightly fibrillar eosinophilic cytoplasm. Their nuclei are oval with stippled chromatin and occasional 1-3 variably sized nucleoli. Anisocytosis and anisokaryosis are moderate,and there are 2 mitotic figures per 10 high power fields. The

polygonal cells have variably discrete borders and a moderate to abundant amount of eosinophilic cytoplasm. Nuclei are oval to irregular with stippled chromatin and 1-2 large, prominent nucleoli. Anisocytosis and anisokaryosis are moderate, and there are scattered mitoses within this population. The overlying mucosa is entirely ulcerated and replaced by a thick band of eosinophilic necrotic debris admixed with abundant degenerate neutrophils and

pyknotic nuclear debris. Subjacent to the ulcer, there is marked granulation tissue formation, and admixed amongst the granulation tissue are frequent individual or small clusters of highly pleomorphic epithelial cells. These are typically polygonal with variably discrete cell borders, and often resemble those scattered cells described in the deeper neoplastic mass. Sections of tissue from the orad and aborad margins of the intestinal segment show no evidence

of a neoplastic population. There is mild lymphocytic and plasmacytic inflammation in the lamina propria.

**Histological diagnosis:**

A. Hepatitis, nonspecific reactive, mild, with hepatic cord atrophy and sinusoidal dilation, hepatocellular degeneration, mild, and nodular hyperplasia, mild, liver.

B. Consistent with enteritis, lymphocytic and plasmacytic, mild, intestinal margins.

C. Consistent with sarcoma with atypical polygonal cell population, intestine.

**Comment:**

The mass within the intestinal wall is largely composed of a well-differentiated sarcoma that is most suggestive of either a leiomyosarcoma or a gastrointestinal stromal tumour (GIST). However, there is also a population of highly atypical polygonal cells that are admixed throughout and somewhat resemble pleomorphic epithelial cells. There is no overt epithelial mass present within these sections, however, I am concerned that there are potentially either

two distinct neoplastic processes occurring in the same region (the sarcoma within the wall with a carcinoma developing from the ulcerated mucosal surface), or that there is a subset of the sarcoma cells that are much more highly pleomorphic and likely aggressive than their counterparts, possibly arising from a mesenchymal to epithelial transition. Regardless of origin, I would be concerned that this population of cells, albeit a very small proportion of the overall mass, would be more likely to have an aggressive behaviour with a higher potential for metastasis. Additional immunohistochemistry would be suggested to further characterise the neoplastic cells. This would help to confirm either a leiomyosarcoma versus a GIST for the majority of the mass, and may help to characterise the population of pleomorphic cells present in this tissue. Please

call if you would like to request additional immunohistochemistry (panel of 4 antibodies).

Case 24

**Clinical History:**

Intestinal mass - previously aspirated as likely carcinoma. At distal duodenum at point of duodenal colic ligament. Resected with anastomosis - whole portion submitted. No enlarged lymph nodes but also biopsied intestinal lymph node.

**Gross description:**

A: Intestinal mass. A beige brown section of tubular tissue measuring 60x25x20mm. Representative sections taken.

B: Mesenteric lymph node. A beige irregular wedge of tissue measuring 5x3x3mm. Embedded whole. All tissue used. Tissue fixed further prior to processing.

**Histological description:**

A. intestinal mass (3 sections). Extending from the widely ulcerated mucosa, extensively infiltrating the submucosa, muscular wall and extending into the mesenteric adipose tissue is a poorly demarcated, infiltrative, moderately to poorly cellular neoplastic mass. Neoplastic cells form tubular structures, which are often ectatic and filled with mucoid material and small nests within abundant fibrovascular stroma (desmoplasia). Individual neoplastic cells are polygonal

with indistinct cell borders and moderate amounts of amphiphilic cytoplasm. Nuclei are round to oval with coarsely stippled chromatin and often multiple small to medium-sized magenta nucleoli. Mitoses are 37 in 10 high power fields. There is marked anisocytosis and anisokaryosis. Neoplastic cells frequently extend to within 1mm of the mesothelial lining. No neoplastic cells are

evident in sections from the proximal and distal surgeon-cut edge.

B. Mesenteric lymph node, incisional biopsy (1 section). This section represents a wedge through the lymph node cortex and medulla. The parenchyma is diffusely well-organised. Cortical and medullary sinuses are moderately expanded by increased numbers of sinus histiocytes.

**Histological diagnosis:**

A. Adenocarcinoma, intestinal mass.

B. Sinus histiocytosis, moderate, mesenteric lymph node.

**Comment:**

Consistent with the FNA results, the submitted intestinal mass represents an adenocarcinoma. Neoplastic cells extend into the mesentery and frequently extend to close to the mesothelial lining of the intestine and mesentery. This tumour would be expected to have a high potential for intracoelomic spread, as well as lymph node and distant metastasis, though no evidence for metastasis is observed in the submitted incisional biopsy from a mesenteric lymph node. No neoplastic cells are evident in the examined sections from the proximal and distal surgeon-cut edge.

Case 25

**Clinical History:**

IBD. Abdo exploration revealed a moderate size mass on the descending duodenum caudal to the pole of the right pancreas followed by a large ulcerated mass at the duodenal flexor, attached to the mesenteric root, involving a large mass that could be the mesenteric lymph nodes.

**Gross description:**

Duodenal mass. A beige grey section of tubular tissue measuring 225x60x40mm with multiple masses measuring up to 40x40mm. Representative sections taken.

**Histological description:**

Descending duodenum (4 sections). These sections comprise an expanded intestinal wall. There is peripheral fibroadipose connective tissue of the mesentery. Remnants of the muscular layers are present in some areas but this layer is frequently infiltrated by a neoplastic cell population (described below). The submucosa, muscularis mucosa and mucosa contain remnants of expected architecture and cells but mostly these areas are expanded by clear space and

lymphatic vessels are often widely patent (oedema). There are focally numerous neutrophils within areas which appear ischaemic. There are multiple foci of brightly eosinophilic cellular and proteinaceous debris sometimes forming dense coagulae. Within these areas is a population of round cells which is often necrotic but where viable can be seen to have moderately distinct cell margins and sparse amounts of palely eosinophilic cytoplasm. Nuclei are round to oval to irregular and have coarsely clumped chromatin and mitoses are 11 per 1x400 field views. The nuclei of the neoplastic cells are up to twice the size of adjacent erythrocytes. The luminal aspect of the necrotic mucosa is encrusted with numerous colonies of coccoid bacteria. Excision edges (2 sections). These sections have long slender villi on the luminal aspects. The proprial stroma is moderately expanded by clear space and lymphatic vessels are widely patent

(oedema). There is modest oedema also of the submucosa and muscularis layers. Some blood vessels are widely patent and contain moderate aggregates of marginating neutrophils.

**Histological diagnosis:**

Compatible with lymphoma, large cell, high grade; with necrosis, multifocally marked, duodenum.

**Comment:**

The histological appearance of the mass described surgically is consistent with a focally marked area of necrosis and oedema of the gut wall. In some areas a viable cell population which appears to be of large round cell with high mitotic activity is recognised and these have histological features of lymphoma. There are numerous areas in which there is granulocytic inflammation which may be neutrophilic although multiple foci of coagulated brightly eosinophilic protein also give the impression of an eosinophilic inflammatory response. This mass appears to have been entirely excised with no neoplastic cells apparent in the

excision edges. It cannot be ruled out however that the tumour will have spread by lymphatic vessels to mesenteric lymph nodes.

Case 26

**Clinical History:**

Vomiting, weight loss, reduced appetite over last 2-3 months. Jejunal mass resected at sx. Splenic nodule noted. Splenectomy performed.

**Gross description:**

A: A beige grey section of tubular tissue measuring 180x50x40mm with a mass measuring 65x40mm. Representative sections taken.

B: A brown irregular wedge of tissue measuring 25x25x10mm with a mass measuring 10x10mm. Representative sections taken. Tissue fixed further prior to processing.

**Histological description:**

A. Jejunum (3 sections). Arising within the jejunum and effacing the mucosa is an infiltrative, poorly demarcated mass that largely fills the lumen. The mass consists mainly of collagenous stroma with scattered aggregates of a cellular infiltrate. The infiltrates consist mainly of eosinophils and suspected lymphocytes and occasional plasma cells. Very rarely, there are individual large polygonal cells with pale cytoplasm surrounding oval to irregular large nuclei with stippled to vesicular chromatin. A mixed cell infiltrate that appears to consist of lymphocytes, plasma cells, and eosinophils continues into the submucosa and muscularis. In one of the sections, there is a rare, single cluster of remnant crypts, that appear to be lined by a single layer of epithelial cells with a single crypt that is markedly dilated and contains abundant degenerate

neutrophils and eosinophilic debris. Transverse sections from the orad and aborad margins are examined with no evidence of neoplastic cells present.

B. Spleen (2 sections). Within the splenic parenchyma is a single, well demarcated, unencapsulated, proliferative nodule that has been fully excised with splenectomy. The mass is composed of multiple discrete and variably well developed lymphoid follicles supported by a moderate amount of congested stroma. Many of the lymphoid follicles retain recognizable architectural features including a germinal centre and surrounding mantel-cell cuff.

**Histological diagnosis:**

A. T-cell lymphoma, large-cell, intermediate grade, transmural, jejunum.

[Original diagnosis: A. Consistent with poorly differentiated neoplasm, jejunum.]

B. Lymphofollicular hyperplasia, nodular, spleen.

**Comment:**

The mass within the jejunum is poorly cellular and is mainly composed of abundant stroma. There are rare individual polygonal cells that raise the

possibility for an adenocarcinoma, but these cells are very sparse. Alternatively, I considered a possible lymphoma as there appear to be clusters of round cells present throughout the stroma, however, lymphoma typically does not elicit a stromal reaction. In light of the poor cellularity, I am trimming in additional sections to try to look for additional diagnostic tissue. An addendum will be sent with results of additional tissue. If results are similar, additional immunohistochemistry would be recommended to further characterise the cells

present in the tissue, and this would help to highlight any atypical epithelial cells present within the tissue that would be indicative of an adenocarcinoma. Please call if you would like to request additional immunohistochemical staining (panel of 5 antibodies). The nodule within the spleen is consistent with an area of lymphoid hyperplasia. This is a benign nodule for which splenectomy is expected to be curative.

**Immunohistochemical description**

The majority of the infiltrating round cells show weak to strong cytoplasmic and membranous immunoreactivity for CD3. Virtually all of the cells (round cells and eosinophils) as well as scattered individual larger round to polygonal cells show strong positive membranous immunoreactivity for CD18. There are scattered individual or small clusters of small, non-neoplastic round cells that are positive for Pax5 and CD20, and rare cells positive for MUM1.

Very rare individual cells are positive for CD117. The occasional entrapped well differentiated crypt-like aggregates are lined by cells with strong positive membranous immunoreactivity for cytokeratin.

**Final Comment:**

Additional tissue sections were trimmed in to evaluated and were fairly similar to the first sections. There is a moderately cellular infiltrate with cells arranged in loose clusters separated by a moderate to occasionally abundant amount of collagenous stroma. The cellular infiltrate is mainly composed of poorly differentiated round cells and abundant eosinophils. The round cells have indistinct borders with a scant to moderate amount of pale cytoplasm. Nuclei are round to oval and range from hyperchromatic to having coarsely clumped chromatin. Some nuclei are up to 2 erythrocytes in diameter and occasional individual nuclei are slightly greater than 2. Anisocytosis and anisokaryosis are mild to moderate, and there are up to 6 mitotic figures in a single high power field. Admixed amongst these round cells and eosinophils are occasional individual larger round to polygonal cells, and rare clusters of slightly irregular polygonal cells. Based on additional sections, the tissue was slightly more suggestive for lymphoma as the bulk of the cellular infiltrate appeared to be round cells. However, there were rare entrapped epithelial structures and well as scattered individual polygonal cells, still raising the possibility for an adenocarcinoma. Based on these findings, additional immunohistochemistry was pursued. The majority of the infiltrating cells (the round cells described above) exhibit weak to strong CD3 staining, indicative of a T-cell origin, and consistent with a T-cell lymphoma. (These cells are also positive for CD18, consistent with a leukocytic origin.) There are occasional scattered individual B-cells that are positive for Pax5 and CD20, consistent with a small inflammatory infiltrate. Similarly there are rare plasma cells (MUM1 positive). The scattered individual larger round to polygonal cells are positive for CD18, consistent with a leukocytic origin, and likely represent an infiltrating macrophage population, consistent with an inflammatory reaction. There are scattered well differentiated crypt-like structures but embedded deep within the mass and no longer associated with the mucosa that are positive for cytokeratin, indicative of an epithelial origin. No other cells throughout the mass are positive for cytokeratin. This suggests that these crypts are likely displaced from the mucosa and have become entrapped within the neoplastic and inflammatory mass, but there is no evidence of a neoplastic or proliferating epithelial population. The rare clusters of more polygonal cells identified on the HE stains do not stain with cytokeratin, and these likely represent entrapped and displaced neurons within ganglia.

The immunohistochemistry confirms that there is a fairly mixed cellular infiltrate throughout these sections of the jejunal mass. The HE findings in conjunction with the additional immunohistochemical findings are consistent with a T-cell lymphoma. There are some unusual features including the highly mixed nature of the infiltrate as well as a more robust stromal reaction than is typical for lymphoma. Likely, many of these changes are secondary to ulceration and secondary inflammation. Given the slightly atypical features, I have shown these tissue sections and immunohistochemistry to two additional patholgists and we are all in agreement that the findings are consistent with lymphoma. Based on the mitotic rate, much of this mass would be considered low-grade, though there are a few fields that are consistent with an intermediate grade designation (6-10 mitoses per high power field).

Although the mass has been fully resected, and there are no neoplastic cells present at the sections from the orad and aborad margins, this neoplasm is expected to be progressive, and continued monitoring of lymph nodes as well as additional viscera would be warranted.

Case 27

**Clinical History:**
Jejunal mass excised with 4-5cm of normal intestine on oral and aboral ends. Mesenteric lymph node was also excised and submitted.
**Macroscopic Description:**
A:  A beige grey section of tubular tissue measuring 110x50x35mm with a mass measuring 40x40mm. Representative sections taken. 
B: Mesenteric lymph node. A beige brown piece of ovoid shaped tissue measuring 10x8x5mm. Bisected. All tissue used. Tissue fixed further prior to processing.
**Microscopic Description:**
A. Jejunum (5 sections). Within the submucosa and tunica muscularis there is a non-encapsulated and bluntly infiltrative neoplasm. Neoplastic cells are arranged in streams and interlacing bundles, supported by a fine fibrovascular stroma. Neoplastic cells are spindle shaped with a moderate amount of eosinophilic cytoplasm and indistinct cell borders. Nuclei are oval to elongated with finely stippled chromatin and one nucleolus. There is moderate anisocytosis and anisokaryosis. 3 mitoses are observed in 10 high power fields. Within the mass there are multiple extensive areas of necrosis with aggregates of fibrin and karyorrhectic debris. No obvious neoplastic tissue is observed within sections through the surgeon-cut tissue borders of the jejunum.
B. Mesenteric lymph node (2 sections). The lymph node architecture is preserved. Within the cortex there are well organised follicles. Medullary sinuses are moderately expanded by erythrocytes and macrophages, some of which contain haemosiderin.
**Immunohistochemical Description:**
Neoplastic cells exhibit cytoplasmic labelling for vimentin and approximately 40% of neoplastic cells exhibit cytoplasmic labelling for smooth muscle actin. Neoplastic cells do not exhibit labelling for S100 or CD117(kit).
**Final Diagnosis:**
A. Leiomyosarcoma, jejunum.
B. Sinus histiocytosis, multifocal, moderate, mesenteric lymph nodes.
First Comment:
The mass within the jejunum is a spindle cell tumour, arising within the tunica muscularis. The main differential diagnoses for this tumour are a leiomyosarcoma or a gastrointestinal stromal tumour. A peripheral nerve sheath tumour is a potential but less likely differential diagnosis. Further characterisation of the neoplastic cells using immunohistochemistry can be performed on request (4 antibodies). Based on the examined sections, this tumour appears to have been completely excised. The mitotic count of this tumour is low and overall metastasis from this site appears less likely. Nevertheless leiomyosarcomas, as well as gastrointestinal stroma tumours (GISTs) have some potential for metastasis and therefore continued monitoring of the patient is recommended. No obvious neoplastic tissue is observed within the mesenteric lymph node.
Addendum:
The neoplastic cells exhibit expression of vimentin, consistent with a mesenchymal origin. There is labelling for smooth muscle actin in approximately 40% of neoplastic cells. This supports a leiomyosarcoma. The neoplastic cells do not express CD117 and there is therefore no evidence for a gastrointestinal stroma tumour (GIST). Furthermore there is no expression of S100 and a peripheral nerve sheath tumour is therefore not likely. Leiomyosarcomas have some potential for metastasis but, given the low mitotic count, this is less likely in this case.
